# Malarial Hæmoglobinuria

**Published:** 1892-01

**Authors:** H. McHatton

**Affiliations:** Macon, Ga.


### MALARIAL HÆMOGLOBINURIA—ANSWER TO DR. PARHAM.

I regret that absence from the city prevented my answering Dr. Parham's article last month. The doctor states he is not aware that the above disease is not a hemorrhage, no matter how confident I may speak on the subject; that it has all the characteristics of a hemorrhage discoverable without the aid of a microscope; in which he is perfectly correct, and for the same reason the disease has been erroneously treated as a hemorrhage for many years. If there is any way that an absolute differential diagnosis can be made between hæmaturia and hæmoglobinuria without the aid of the microscope, I wish the doctor would publish the secret, as he is the only one that possesses it. Until then, as he acknowledges that he has no availed himself of the only known way in which the diagnosis can be made, it seems only reasonable that he should allow a certain amount of credence to the reports of numberless good scientists who have made this examination, and without a dissenting voice pronounced it a hæmoglobinuria.

The doctor takes me to task for using preventive medication, and for treating a disease before it exists. I had hoped that the following, as it occurs in my original article, was explicit enough to be understood by a first-course student, as I can't see how a disease can be treated before it is diagnosed: "I should advise, as soon as the case is diagnosed, a sufficient dose of quinine to cinchonize the patient, say thirty grains in solution, or its equivalent hypodermically."

I can say now, after some years' experience, that preventive medication is of great value in the disease. The doctor asks what malaria is. He might as well ask what scarlet fever or typhoid fever are, or, better still, tell me what morbid secretions are. The *modus operandi* of quinine, as has been observed by competent scientists, is to cause the disappearance of the intra-corpuscular bodies in the red blood corpuscles which occur at the beginning of the paroxysm, and probably go on to the complete destruction of the invaded corpuscle when not influenced by medication. This action has been observed since my original article. The number of corpuscles invaded and destroyed before any medication is given probably accounts for the invariable fatality of the pernicious cases of this disease. The doctor may deny that quinine has any control over malaria; that is his opinion; but in the present status of the question it will require some new facts to shake the belief of the profession in this action of quinine, and without such facts there can, of course, be no argument on that issue.

In regard to ergotole, I am sure that I do not know what the doctor intended to say. He did say, "I control the hemorrhage and alter the abnormal condition of the blood with the following: Ergotole, tinct. iron, aa gtt. x." Which is an absolute assertion with, as far as I can see, no opening for escape. The evacuant pleasantry was the result of a typographical error in the doctor's first article. I asked the doctor to furnish me with some statistics of this disease, accurately observed, before the publication of my original paper. He furnishes me with some recollections of the disease, of no scientific value, but still presenting some peculiarities.

He has had one hundred cases with only one death, and that, we are led to infer, died of heart disease, consequently he has had ninety-nine consecutive recoveries. All of them of the continued type of fever.

Other observers look upon the continued type as the rare form of the disease. His treatment varies in no essential from the treatment that has been used for the past fifty or one hundred years. In all available statistics the mortality is from thirty per cent. up, as the course of the disease and the result of the treatment is so decidedly at variance with all other observers, it would lead one to suppose, taking these two factors into consideration, that there might have been some error in diagnosis.

There is a late paper by W. T. Prout, M. D., C. M. Edin, goverment officer, Gold Coast, W. Africa, (*Lancet*, Aug. 1st, 1891) on malaria, that is of interest to all students of this disease, from which I quote as follows, as bearing special reference to the subject in question.

"The third type is the hæmoglobinuric form, which is fortunately the rarest, as it is the most severe and most dangerous. It rarely occurs in Europeans who are in robust health, but in those who have become debilitated and anæmic from repeated attacks of fever, or from mental worry or alcoholic or other excess. I am inclined to associate it more particularly with any cause which interferes with the action of the liver, as I have seen it occur several times in beer-drinkers. The onset is sudden, accompanied it may be with slight shivering. Vomiting commences early, and is a prominent and intractable symtom throughout. At first it consists of bile, and laterly merely of the liquids imbibed, mixed with dark green shreddy particles. The tongue and breath are foul, and there is great thirst. The skin, conjunctivæ, and buccal mucous membranes are of a bright canary-yellow color. The temperature need not necessarily rise very high, and may even fall to normal, though the other symptoms persist. If after this there is a constantly rising temperature without remission, the prognosis is unfavorable. The state of the urine early attracts the patient's attention. In the worst cases it is a dark porter-like color, is almost syrupy in consistence, frothes easily and stains the sides of the vessel containing it a bright crimson. At this stage boiling and nitric acid show the presence of a considerable quantity of albumen. A copious deposit forms on standing, which is seen to consist of pigment granules and pigment casts from the kidneys. A few blood-cells may be present, but they are not common. The quantity of urine is usually diminished. Recovery may take place, and in this case the general symptoms improve, and the urine gradually becomes less high colored, increases in quantity, and becomes loaded with urates. Convalescence is naturally slow, and departure from the coast is usually indicated. On the other hand the symptoms may become aggravated; the urine diminishes in quantity, although the color generally improves, and total suppression may take place. Death in these cases results in from three to five days from uræmia and exhaustion."

With reference to the time of occurrence of these bodies, it may be observed generally that the intra-corpuscular forms were present before the paroxysm and while the temperature was rising, and usually disappears under treatment.

The effect of quinine in the cases in which I was able to make several examinations, appeared to be the disappearance of the intra-corpuscular bodies.

The efficacy of quinine in the treatment of malaria is a point on which some doubt has recently been thrown, but the more I have to do with this disease the greater becomes my confidence in the judicious administration of quinine, which, so far as I am able to judge, is the only drug which can be relied upon to cure malarial fever.

There is no doubt that malarial fever, like many other diseases, has a tendency to be self-limiting, but it does not follow that quinine is of no use because certain cases recover without the administration of any drug. I myself have treated many cases of intermittent fever among coolies in Mauritius with a simple infusion of quassia, or sometimes have given them no treatment, and have found the fever gradually disappear. That, however, only holds good when the vitality of the tissues, and principally the blood tissue, is sufficient to cope with the malarial poison; but where the vitality of the body is lowered by any cause, or where the quantity of poison absorbed has been very great, I find quinine can be relied upon to cut short the attack.

Quinine can only be regarded as a poison to the malarial organism, killing it and preventing its further development. There are no doubt certain cases in which quinine fails entirely; but they are rare, and I believe are not pure cases of malarial fever, but probably connected with some obscure complication often connected with the state of the intestines.

It appears to me to be a reasonable position to take up that if quinine is antagonistic to the malarial poison, the administration of small doses, say two or three grains daily, will be inimical to the development of the organism, just as a two per cent. solution of carbolic acid retards the growth of the spores of the anthrax bacillus, although a five per cent. solution is required to kill them. I can quote only one case, namely my own, which of course has little weight by itself. For a period extending over several months I used small doses of quinine without any evil effect, and with the result of keeping me fever free, while after I ceased to take it, I suffered from a mild intermittent within three weeks.

Should Dr. Parham offer any new points on the pathology, clinical history or treatment of this interesting and dangerous disease, I shall be glad to continue this discussion, otherwise I beg to decline. Should any of the readers of this journal deem it of sufficient interest, I shall be glad to furnish them with copies of my original article, a few of which I still have on hand.

